# A Journey Into the Unknown: PhD Students in a European Training Network on Age-related Changes in Hematopoiesis Conduct Their Project During a Global Pandemic

**DOI:** 10.1097/HS9.0000000000000763

**Published:** 2022-07-29

**Authors:** Christina Pitsillidou, Sandra Alonso-Rubido, Andrea Ávila-Ávila, Mari Carmen Romero-Mulero, Agata Labedz, Athanasios Oikonomou, Ludovica Proietti, Maria Eleni Psychoyiou, Clara Tellez-Quijorna, Maniriho Hillary, Eirini Sofia Fasouli, Guillermo Fernández-Rodríguez, Natalia Giner-Laguarda, Natalia Skinder, Chiara Taroni, John Strouboulis, Eleni Katsantoni, Antonella Ellena Ronchi

**Affiliations:** 1 Dipartimento di Biotecnologie e Bioscienze, Università degli Studi Milano-Bicocca, Milano, Italy; 2 FlowMetric Europe, Milan, Bresso, Italy; 3 Diagenode SA, Ougrée, Belgium; 4 Institute Curie, Orsay, France; 5 Centre National de la Recherche Scientifique UMR3348, Centre Universitaire, Orsay, France; 6 Max Planck Institute of Immunobiology and Epigenetics, Freiburg, Germany; 7 Fondazione Tettamanti, Centro di Ricerca Tettamanti, Centro Maria Letizia Verga, Monza (MB), Italy; 8 Dipartimento di Medicina e Chirurgia, Università degli Studi Milano-Bicocca, Monza (MB), Italy; 9 Institute of Medical Biochemistry, University of Veterinary Medicine, Vienna, Austria; 10 Molecular Haematology, Comprehensive Cancer Centre, School of Cancer and Pharmaceutical Sciences, King’s College London, United Kingdom; 11 Team of Epigenetics in Normal and Abnormal Hematopoiesis, CRCM (Centre de Recherche en Cancèrologie de Marseille), France; 12 Department of Human Molecular Genetics and Biochemistry, Sackler Faculty of Medicine, Tel Aviv University, Tel Aviv-Yafo and Schneider Children’s Medical Centre of Israel, Petach, Tikvah; 13 Basic Research Center, Biomedical Research Foundation, Academy of Athens, Greece; 14 Department of Biology and Biotechnology “Charles Darwin”, Sapienza University of Rome, Italy; 15 Department of Cellular and Molecular Biology, Centro de Investigaciones Biológicas Margarita Salas, Madrid, Spain; 16 European Research Institute for the Biology of Ageing, University Medical Center Groningen, University of Groningen, Netherlands; 17 Institut de Génétique et de Biologie Moléculaire et Cellulaire (IGBMC), Institut National de la Santé et de la Recherche Médicale (INSERM) U1258, Centre National de la Recherche Scientifique (CNRS) UMR 7104, Université de Strasbourg, Illkirch, France

The age-related changes in hematopoiesis (ARCH) project is part of the Innovative Training Network (ITN) of the Marie-Sklodowska Curie Actions (MSCA) program, which provides doctorate training of excellence based on the exchange of ideas and competencies from the academic and private sectors.^1^ The ARCH project intends to outline hematopoietic stem cell (HSC) alterations with aging, how hematopoietic cell individuality is controlled at the transcriptional and epigenetic levels in normal hematopoiesis and in leukemias, and understand the crosstalk between intrinsic and extrinsic indications that support the proliferation of preleukemic and leukemic cells within the hematopoietic niche.

We are 15 PhD students funded by this network, based around Europe, and our common aim is to understand functional changes in the hematopoietic system with age, how these changes link to the development of age-associated diseases and in parallel work towards the development of new treatments.^2^ Our projects kicked off just when the severe acute respiratory coronavirus-2 (SARS-CoV-2) emerged. Two and a half years later, SARS-CoV-2 continues to infect millions of people and has taken the lives of at least 6 million people worldwide.^3^ The COVID-19 outbreak brought along social isolation and feelings of uncertainty to everyone around the world, including doctorate students.^4,5^ Ironically, our projects have been more relevant than ever, as the pandemic has highlighted the important relationship between age-related changes in hematopoiesis and disease severity. Below, we aim to discuss the timeline of the ARCH project throughout the pandemic and how we managed to courageously pull through the hardships of doing research during a global pandemic within different settings (academia/institutes and industry). We provide recommendations to future PhD students on how to manage their PhD projects during global emergencies.

## PANDEMIC TIMELINE OF ARCH

**Figure 1.**
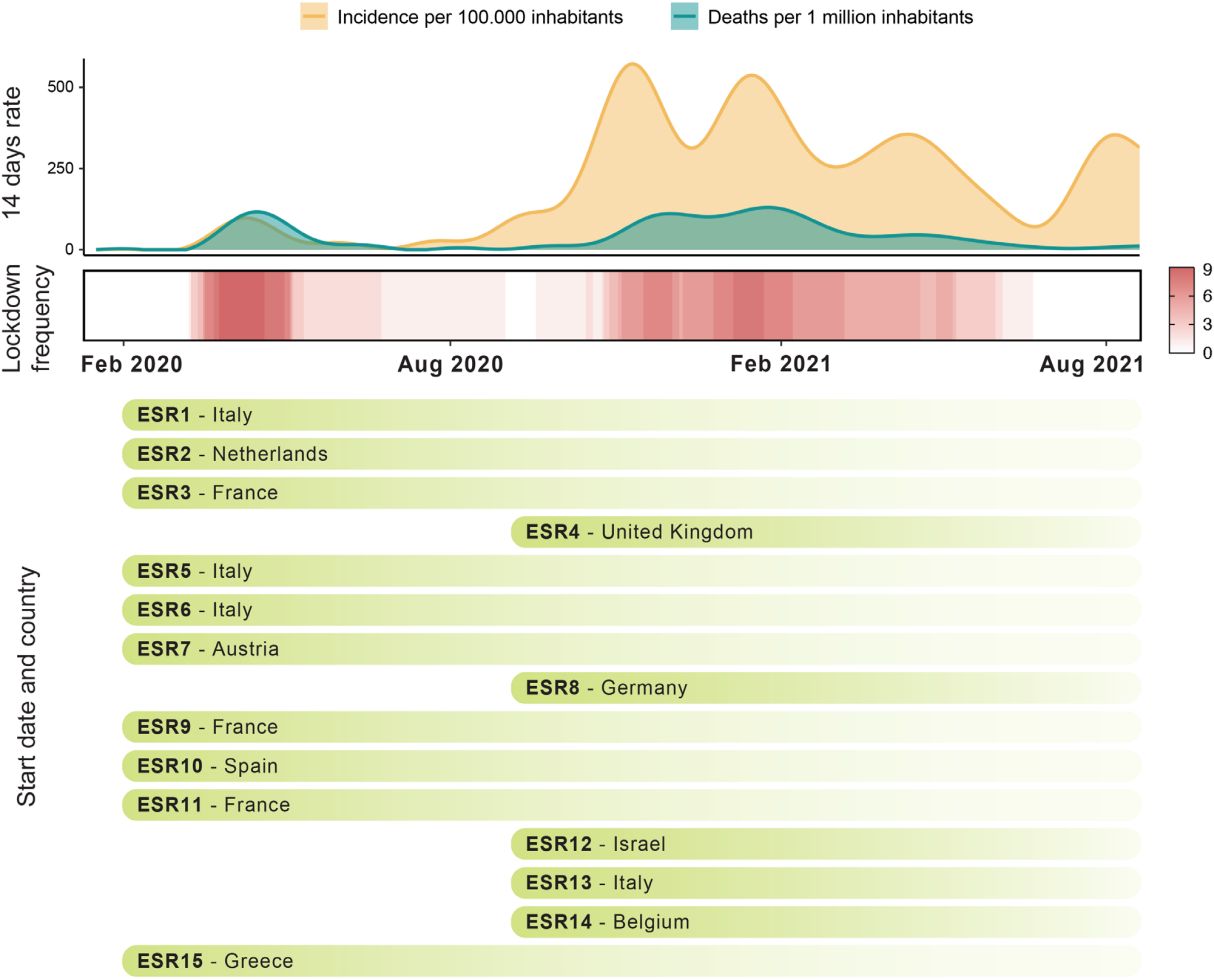
**Timeline of the ARCH project during the COVID-19 pandemic.** Average 14-day notification rates of newly reported COVID-19 cases per 100,000 inhabitants and COVID-19 related deaths per 1 million inhabitants in the participating countries of the ARCH project are represented during the first year and a half of the pandemic in Europe (top graph). By fall 2020, there is an all-time high of the infection and death rates. The lockdown frequency considered as the daily number of ARCH participating countries under measurements of lockdown is shown in a heat-map along the same period (middle graph). Starting time and country of the beneficiary institution where each early stage researcher (ESR) performs the PhD project is shown in bars (bottom graph). These data have been collected from official governmental reports from each country. The lockdown definition and concrete measures differ between countries and regions within the countries.

The rapid spread of SARS-CoV-2 at the beginning of 2020 in Europe led the World Health Organization to declare a global pandemic on March 11, 2020. The ARCH opening conference, planned for the same month was canceled. At the same time, the first lockdowns were put in place around Europe, while most of us were just starting our new project or were moving to a different country to begin. Some of us started on this project during or after the first lockdown, causing further delays, which led to a multiphasic pattern of recruitment (Figure 1). During the first lockdown, we individually set up our strategies to progress our research projects at home by either reading scientific literature, writing review papers,^6-9^ or planning experiments for when we would eventually return to work. By summer 2020, many of us began to slowly get back to the laboratory in shifts or for a limited time to avoid overcrowding of spaces. Times were still uncertain and the pandemic was of great concern to the global scientific community. With the whole world waiting for vaccine developments to stop the pandemic, our slow training process commenced. However, by fall, new lockdown measures were set against a new surge of infections and deaths (Figure 1). This second lockdown stretched until Christmas, marking the completion of our first year with minimal hands-on training and very limited experimental progress. This brings us to today, where most of us have entered the final year of our projects and it seems that COVID-19 is here to stay.

## COVID-19: PHDS INTO THE UNKNOWN

Engaging in a PhD project on its own is stressful and overwhelming. The main objectives when beginning a 3-year PhD project are to develop new laboratory, academic, and research-based skills, yet we were unable to advance on any of these. Close and continuous supervision is required to meet these goals. Communication with supervisors at the beginning of our PhDs, which coincided with the pandemic, made our start more challenging than expected. Coming out of isolation in the summer of 2020, we did not expect those upcoming months would continue to affect our projects and to make us experience how vulnerable we are.

Working hours were adjusted to allow for social distancing, while meetings were still being held online. Even when we were physically at the laboratory, there was an inability for close supervision due to safety restrictions. In addition to communication challenges, technical difficulties were also at play during the first lockdown. For example, students that had already begun to breed mice a few weeks before the lockdown were forced to interrupt their experiments and redirect their projects as it would take too much time to re-establish mouse breeding. The high demand for equipment, reagents, and consumables for the advancement of COVID-19 research and diagnosis added to these difficulties and made it challenging for us to obtain the necessary consumables for our research. Reagents and consumables were not the only shortage, but also sample availability. As our projects are based on hematology, many of our experiments require fresh samples from healthy or diseased participants to progress. Such fresh materials were merely impossible to find as clinics halted unnecessary visits and hospitals gave sole priority to COVID-19 patients. Again, this inevitably led to the redirection of some projects and added to the uncertainties and delays of the projects.

Some institutions acknowledged the loss of time for many PhD students and offered time extensions for thesis submissions. In some institutions, time extensions were only applicable to students that were enrolled in their second and third year, a situation that we consider is not justified as the pandemic had a strong impact on all PhD projects regardless of the year of enrollment. However, those that were offered an extension were offered no additional financial support. This made us feel that our PhD training status was overlooked and shows once more that PhD positions are a precarious form of employment. As these are typically 3 to 4 year training periods, where PhD students need to provide evidence of strong scientific skills, not being able to work is a major limitation not only to obtain the PhD degree, but also for the success in making next step in the career.

COVID-19 repercussions created disruptions also to life science companies. Twenty percent of ARCH students work in the private sector. Companies had to adapt to the COVID-19 pandemic to survive, thus made COVID-19 research and treatment innovation their uppermost priority. Industry supervisors were understandably busy involved in clinical research projects to meet client requests. Lockdown measures stalled many investigations, having a domino effect on doctorate projects based there. At the same time, the detachment of industry PhDs from research institutions and universities negatively affected their learning progress.

The main scientific activities were mostly virtual and we missed the stimulus of having an audience present, while many such events were inadequately organized and prone to technical glitches. Virtual learning left gaps in milestone competencies like collaboration and the development of a critical approach to research questions and results. However, we are thankful for online platforms. Regardless of the complexity of our position, many of us took advantage of the lockdown by completing online courses and taking part in virtual conferences. Virtual platforms at least allowed us to have some communication and to develop more valuable computational skills than ever before, which were also applied to our projects.

## WHAT PHD STUDENTS CAN DO DURING GLOBAL EMERGENCIES?

Doctorate students aim to find the answers to complex scientific problems and need to excel in critical thinking and correct decision-making, which already imposes pressure on them. Experiencing external pressures through a global pandemic makes it harder to face the challenges associated with research. Here we offer our advice to students that could be experiencing similar global emergencies:

Mental wellbeing is one of the biggest challenges one will face when conducting a PhD project in such peculiar times. Even under the best circumstances, one cannot be productive at all times. Therefore, we recommend PhD students to avoid harsh self-criticism if they are lack motivation. We strongly encourage PhD students to share feelings with their peers and colleagues in the same consortium or institution and thus to be open and talk about all problems. We created a group chat where we shared our thoughts and worries. This exercise made us realize that we were not alone and others could relate to our emotions, which was comforting. It is important to rely on other students and researchers to find personal support. Do not hesitate to reach out to your supervisor for help regarding your mental wellbeing as this can have an impact on your work. Good supervision and communication are crucial for the proper development and success of your project.

To make the most out of a PhD project we recommend that PhD students take advantage of all opportunities and plan. Design experiments to be performed during times when you can enter the laboratory and plan to generate enough data that you can analyze if a new lockdowns arises. We recommend students to start writing anything related to their research field. For example, write the introduction of the PhD thesis, methodologies that will be used, write a review article, and so on. PhD students will have to write at some point, so they should not hesitate to start even with no results or experience in the field they have just embarked on. Additionally, online seminars can be followed as well as online courses, for example to develop new computational or experimental skills.

## CONCLUSION

Despite all the difficulties, we feel fortunate to be part of the ARCH MSCA ITN program. In December 2021, after 2 years of travel restrictions, we finally met for the first time in person in Rome. Here students and supervisors used this opportunity to begin collaborations, present projects, discuss, and overcome limitations.

The COVID-19 pandemic continues to have a significant impact on scientists. PhD students completing their projects throughout the global pandemic have suffered immense isolation and anxiety. Despite recurring delays throughout our project, we feel we have emerged as efficient workers, excellent planners and have developed strong social skills virtually and in person. Those experiencing similar global emergencies should share their experience with peers and colleagues, and find ways to efficiently transit towards effective solutions.

Finally, we hope that PhD students experiencing such situations are acknowledged for the time lost and hope that funding agencies recognize that time extensions should be accompanied by financial compensation.

## DISCLOSURES

The authors have no conflicts of interest to disclose.

## SOURCE OF FUNDING

This project has received funding from the European Union’s Horizon 2020 Research and Innovation Program under the Marie Skłodowska-Curie grant agreement no. 813091 (ARCH, age-related changes in hematopoiesis).
